# Analysing the engagement with pandemic preparedness, prevention and response in selected English language political manifestoes in 2024

**DOI:** 10.1080/16549716.2025.2559453

**Published:** 2025-09-19

**Authors:** Clare Wenham, Akhila Potluru

**Affiliations:** a Department of Health Policy, London School of Economics and Political Science, London, UK; b LSE Health, London School of Economics and Political Science, London, UK

**Keywords:** Political prioritization, pandemic fatigue, political parties, pandemic prevention, preparedness and response, elections

## Abstract

Despite the devastating impact of COVID-19 and repeated calls for political commitment to health security, our analysis of 43 manifestos from 16 countries and the European Parliamentary elections revealed that only four parties made specific policy pledges on pandemic prevention, preparedness and response, with six providing brief mentions. The vast majority (33 parties) did not mention pandemic prevention, preparedness and response. When referenced, the pandemic was often framed as a rare, one-off crisis or an economic shock rather than a catalyst for systemic health reform. Some parties used it for political critique or validation of past performance, while others framed preparedness in terms of national security or economic resilience rather than public health. In contrast, manifestos overwhelmingly prioritized healthcare system expansion, equity, and access, with a significant emphasis on universal health coverage, mental health, and workforce development. The findings underscore a stark misalignment between global health priorities and domestic political agenda. Political reluctance to emphasize pandemic prevention, preparedness and response appears to be influenced by pandemic fatigue, voter preferences for forward-looking narratives, and institutional incentives favouring short-term tangible outcomes. This persistent neglect of the pandemic in electoral discourse raises concerns about the global community’s ability to sustain momentum for pandemic resilience. We call for stronger engagement between the global health community and political actors to elevate pandemic prevention, preparedness and response as a strategic, cross-cutting priority for future policymaking.

## Paper Context

- **Main findings:** only four party manifestos specifically included pandemic prevention, preparedness and response in their electoral pledges, with the majority not mentioning this at all.
- **Added knowledge:** There is a significant gap between the need for enhanced pandemic prevention, preparedness and response, and the global measures being taken to enhance it, and the realities on the ground that politicians are prioritizing.
- **Global health impact for policy and action:** Global health actors need to engage more in domestic politics and politicians to be able to enhance pandemic prevention, preparedness and response.

## Background

As COVID-19 exposed, the cost of not being prepared as a government for an emerging epidemic is disastrous. Given that we have just lived through what we hope will be the largest pandemic of our lifetime, we presumed that future governments would learn from lessons identified by reviews on COVID-19 and incorporate pandemic prevention, preparedness, and response (PPPR) into their policy priorities. Commitment to PPPR requires political will at the highest echelons of government and not simply left to the domain of public health [1].

In 2024, more than half of the world’s population went to polls, with over 80 countries holding elections. These elections, occurring in the aftermath of COVID-19, unveiled the potential for voting outcomes to influence future PPPR policies. Yet, most people across the globe suffer from pandemic fatigue and want to forget that it happened [2]. As such, prospective parties vying for attention are caught between creating policy pledges to enhance PPPR for the future and wanting to appeal to the popular vote, thereby focusing on the issues that people want to talk about (and do not mention the ones people do not want to talk about, such as pandemics).

Political manifestos serve as key indicators of a party’s policy priorities and are instrumental in shaping political discourse during elections [3]. In terms of health, political manifestos have historically prioritized immediate domestic health concerns: ageing populations, healthcare access, and non-communicable diseases, while largely neglecting systemic issues such as pandemic preparedness [4,5].

We wanted to know whether, in the wake of COVID-19, political parties were seeking to prioritize PPPR in their electoral pledges. A critique of the 2024 general election manifestos in the United Kingdom [6], for example, noted the alarming omission of PPPR and antimicrobial resistance despite the economic and human toll of COVID-19, and we wanted to know how reflective this was of the global electoral landscape.

We undertook a comparative analysis of 43 manifestos from 17 different elections (16 countries and the European Parliamentary Elections) to analyse how political parties framed PPPR in their 2024 election manifestos, and if they did not include them, what health issues they prioritized instead. This study contributes to the broader discourse on the integration of health security into political platforms, providing insights into the political commitment to building pandemic-resilient health systems.

## Methodology

We located manifestos for countries that held elections in 2024 [7], where English is the official language (even if not the primary language) representing geographically and politically diverse systems, and those that had publicly available party manifestos. Only documents explicitly identified as official manifestos or policy platforms reflecting the party as a whole [8] for the 2024 elections were included. By limiting the scope to English-language documents, we focused-on content officially communicated by political parties, or those formally translated by the party themselves (i.e. so the language is accurate to the intended meaning).

Manifestos were gathered through systematic Google searches using the following search string: ‘[Political Party Name] [Country] Manifesto 2024.’ The major political parties running the 2024 elections for each country were found by searching the string: ‘[Country] Election 2024.’ and are found at Table 1.

**Table 1.** Countries and number of manifestos analysed.

England (4)	Wales (1)	Scotland (1)
Ireland (3)	USA (2)	European Parliament (4)
Austria (1)	Iceland (1)	Bangladesh (1)
India (2)	Pakistan (3)	Sri Lanka (1)
Ghana (1)	Namibia (13)	Bulgaria (1)
South Sudan (1)	South Africa (3)	

The primary analytical focus was on mentions of PPPR within manifestos. A comprehensive list of search terms was developed from a review of academic literature and policy documents related to PPPR [Table 2] to locate content relevant to pandemic preparedness in these manifestos:

**Table 2.** Search terms utilized.

Pandemic	Health Security/Security	COVID
Corona	Virus	Prepare
Risk	Resilience	Surveillance
Epidemic	Prevention	Trace
Outbreak	Contamination	Infection
Monitoring		

The utilization of PPPR terminology by each party was then categorized into three groups: ‘briefly mention,’ (general acknowledgement of pandemic preparedness without offering further elaboration) ‘mention specifically,’ (introduction of concrete plans, departments, or protocols dedicated to PPPR) and ‘no mention.’ Following the categorization, thematic similarities across political parties were analysed, identifying recurring themes and patterns in how pandemic preparedness was addressed.

## Limitations

First, many countries were excluded from our analysis because of language barriers, including Japan, Venezuela, France, Mexico, and Belgium. Moreover, we were unable to locate manifestos in a small number of cases. This limitation reflects a recurring challenge in manifesto analysis, as not all parties have publicly available manifestos, often forcing researchers to resort to including alternative [9], non-electoral specific, party documents in research. Moreover, it is possible that our search terms in Google missed official party documents, even if in English, reducing the robustness of the sampling.

## Results

Of the 43 political party manifestos analysed, 4 parties ‘Mention specifically’ PPPR in their manifestos, 6 parties ‘Briefly mention’ PPPR, and 33 parties have ‘No mention’ of PPPR.

Across all 43 manifestos, there is no unified narrative about what the pandemic represented and what the implications are for PPPR. Six manifestos framed the pandemic as a crisis unlikely to recur soon, downplaying the urgency of long-term preparedness. For instance, the UK Conservative Party described the pandemic as a ‘once-in-a-generation pandemic,’ while the Indian Bharatiya Janata Party labelled it a ‘once-in-a-century event.’ Many parties echo this sentiment, using ‘unprecedented’ to diminish the urgency for sustained PPPR. Thirteen political parties viewed it as a temporary economic shock and significant driver of economic strain. The Bangladesh Awami League emphasized that ‘the pandemic devastated economies … around the world,’ while in the USA, Democrats described it as a cause of ‘economic difficulties.’ This folds COVID-19 into pre-existing political and financial discussions, rather than an issue demanding structural PPPR improvements.

Four parties used the pandemic to explicitly critique political opponents, such as the UK Green Party attributing rising inequality and food insecurity to ‘a combination of austerity and poor choices in response to Covid’. Conversely, six parties used the pandemic to bolster their political credibility by emphasizing their effective handling of the crisis. Ireland’s Fianna Fáil cited management of COVID-19 as justification to trust their leadership, while Pakistan’s Tehreek-e-Insaf (PTI) highlighted it was ‘internationally recognized for its effective handling of the COVID-19 pandemic, ranking among the top three globally.’ This rhetoric potentially diminishes the urgency to include explicit PPPR in future policy agendas.

An interesting trend in the manifestos is the framing of PPPR as a geopolitical or economic resilience tool, rather than a public health imperative. For example, U.S. Democrats focus on how the pandemic exposed vulnerabilities in supply chains. The UK Conservatives’ manifesto highlights a ‘National Defence and Resilience Plan’ to enhance security and preparedness based on lessons learned from COVID-19, pivoting to broader issues of self-reliance and protection. Pakistan’s Muslim League Party included mention of PPPR under defence and foreign policy, to protect ‘trade, investment, tourism, and development partnerships,’ emphasizing economic competitiveness, supply chain diversification, and strategic autonomy. In contrast, five parties called for an internationalist approach such as improved cross-border collaboration. The European People’s Party advocates for ‘a comprehensive EU-level approach to preparedness and crisis response,’ while the Renew Europe Group emphasizes ‘new rules on cross-border health threats.’

In the absence of significant focus on PPPR, 29 out of 43 parties proposed expansion of universal healthcare infrastructure, coverage, and workforce. The structural organization of healthcare, towards more localized healthcare delivery, is a recurring theme. Sixteen parties emphasized decentralization to improve rural access and promote equity and community-based healthcare. Sri Lanka’s Samagi Jana Sandanaya seeks to improve healthcare provisions via technological transformation in the sector. Eighteen parties aspired to increase the provision of universal health care/insurance. For instance, South Africa’s African National Congress emphasizes a ‘National Health Insurance to make quality health care affordable and available to all,’ while the Indian National Congress commits to making ‘healthcare universal and free in public health centres.’ Moreover, 27 out of the 43 parties propose improving equity of healthcare, whether this is based on race, gender, income, rural/urban, or age.

Others politicized healthcare: the U.S. Republican Party framed healthcare as a service under threat from immigration and advocated for its preservation for American citizens, and the Scottish National Party sought to protect Scotland’s NHS from Westminster-driven privatization. Specific health measures appeared several times; 12 mentions of preventative healthcare measures (e.g. smoking, vaping) and chronic disease prevention (e.g. childhood obesity). Moreover, vaccine development and vaccination programmes are widely promoted Ghana’s New Patriotic Party; for instance, while Pakistan’s Muslim League (PML) focuses on immunization efforts, including polio eradication. Others focused on reducing waiting lists and expanding medical space. Eleven parties address mental health, particularly among younger populations; for example, the European People’s Party proposed an EU Action Plan for Mental Health.

## Discussion

One of the most striking takeaways is the lack of consistency in how parties frame COVID-19 and whether they address PPPR at all, with 33 manifestos failing to mention it. This represents a missed opportunity to enhance gaps in health system resilience that have been identified. Even between similar political ideologies or within the same country, there is no unified approach to discussing the pandemic, indicating that public health emergencies remain a politically fluid issue, rather than a fixed policy concern.

The cycle of panic and neglect is a familiar story in PPPR, with a tendency towards crisis response over prevention. However, given the extensive impact of COVID-19, it is surprising that political neglect began so rapidly. Political turnover disrupts policy continuity, leading to an ‘amnesiac cycle [10],’ where incoming governments neglect prior commitments and weaken long-term health strategies. In turn, political parties may have deprioritized PPPR, given the framing of COVID-19 as a one-in-a-century event, an underestimation of future planning requirements for PPPR.

Public fatigue of COVID-19 may also have impacted campaign strategy preferences. Behavioural research suggests that voters gravitate towards optimistic narratives and, thus, may resist revisiting the pandemic [11]. Given COVID fatigue has already fuelled political discontent among societies [12], parties may perceive PPPR as politically unappealing [13]. Moreover, acknowledging pandemic preparedness may reopen contentious debates, or highlighting vulnerabilities that remain unresolved, undermining voter confidence. By focusing building up health capacity or economic growth, parties seek to appeal to an electorate that may prioritize recovery and future-oriented initiatives.

Moreover, structural and institutional barriers hinder the prioritization of PPPR. Electoral cycles and media pressures drive politicians to focus on immediate, tangible outcomes [14], such as increasing hospital capacity; reducing waiting times; and addressing the needs of specific demographics, which yield visible results for electoral advantage. This short-term focus, compounded by institutional fragmentation and bureaucratic inertia, diminishes the political capital necessary for large-scale investments in PPPR [13]. We note that investments in such broader health system capacity development can also support PPPR activities, whilst not being expressly framed as such.

National security has received significant attention; however, health security has been conspicuously overlooked. This neglect of PPPR in political party manifestos aligns with broader nationalist tendencies that prioritize immediate domestic concerns over transnational collaboration. Sociopolitical dynamics, including the rise of populist movements, disinformation, and societal divisions, exacerbate these tendencies. Such dynamics erode public trust, making it more difficult for governments to justify the systemic measures necessary for PPPR, particularly those that require international coordination. This resistance highlights the complex interplay among ideology, governance, and public sentiment in shaping health policy priorities.

There also remains a misalignment between the political parties’ priorities, and the narratives of the global health community. The amendments to the IHR; the creation of the pandemic fund; the adoption of a pandemic treaty all seem absent to political manifestos domestically. This gap highlights a systemic issue where PPPR efforts, despite their global significance, struggle to gain traction within electoral agendas. This may well be a failure of communication by those working in global health to engage meaningfully with those in the political landscape and demonstrate its vital importance. However, with the now agreed Pandemic Agreement needing domestic ratification, this could be a significant challenge.

## Conclusion

These findings paint a concerning picture; despite the devastating impact of COVID-19, PPPR remains largely absent from political party manifestos worldwide. While some parties briefly acknowledged the pandemic’s economic and social tolls, few translated these lessons into concrete policy commitments for future resilience. Instead, immediate health concerns such as healthcare access, waiting times, and workforce expansion dominated electoral priorities. This finding suggests a disconnect between the global health community’s push for sustained PPPR investment and the political realities shaped by voter preferences and short-term electoral incentives. If pandemic preparedness becomes a genuine political priority, global health advocates must bridge this gap, making a stronger case for its relevance to economic stability, national security, and long-term governance.

## Data Availability

Data will be made available on reasonable request.
